# Patient Reported Experiences of Receiving Person‐Centred, Nurse‐Led Follow‐Up After Revascularisation for Intermittent Claudication: Secondary Analysis of a Randomised Controlled Trial

**DOI:** 10.1111/jocn.17762

**Published:** 2025-05-20

**Authors:** Sara Tibebe Haile, Mina Olsson, Ronnie Lindstrand, Helena Lööf, Anneli Linné, Unn‐Britt Johansson, Eva Joelsson‐Alm

**Affiliations:** ^1^ Karolinska Institutet, Department of Clinical Science and Education Södersjukhuset Stockholm Sweden; ^2^ Department of Surgery Södersjukhuset Stockholm Sweden; ^3^ Sophiahemmet University, Department of Health Promoting Science Stockholm Sweden; ^4^ Mälardalen University, Division of Caring Sciences, School of Healthcare and Social Welfare Västerås Sweden; ^5^ Department of Anesthesiology and Intensive Care Södersjukhuset Stockholm Sweden

**Keywords:** intermittent claudication, nurse‐led, person‐centred care, PREM, quality of care

## Abstract

**Aim:**

To evaluate the quality of care from the patients' perspective after receiving either person‐centred, nurse‐led follow‐up or standard care after surgical treatment of intermittent claudication.

**Design:**

Secondary analysis of a randomised controlled trial.

**Methods:**

Patients at two centres for vascular surgery in Stockholm, Sweden were randomised to either a person‐centred, nurse‐led follow‐up programme (intervention group) or a standard follow‐up programme with surgeons. During their visits at 4 to 8 weeks and 1 year after surgery, they received the questionnaire Quality from patients' perspective with 28 items. The patients responded to each item from two aspects: (1) how they perceived the quality of received care and (2) subjective importance (how important the care was for them).

**Results:**

A total of 104 of 138 patients at 4–8 weeks and 159 of 193 patients at 1 year after surgery completed the questionnaire. At 4–8 weeks, the intervention group scored significantly higher perceived quality of care regarding five items: receiving useful information about “How I should take care of myself” and “Which nurse were responsible for my care”, “Nurses were respectful towards me”, “Nurses showed commitment/cared about me” and “Easy to get in contact with the clinic through telephone”. At 1 year, the intervention group scored higher regarding two items: “Which nurses were responsible for my care” and “Next of kin treated well”.

**Conclusion:**

Person‐centred, nurse‐led follow‐up as implemented in this study has been shown to lead to a higher perception of quality of care regarding information about self‐care, the experience of being respected, and knowing the care provider responsible for their care. Thus, it could contribute towards improved patient satisfaction without compromising the perception of quality of care regarding other factors such as receiving the best medical care or timeliness.

**Implications for the Profession and/or Patient Care:**

This study addresses how patients with intermittent claudication, who underwent revascularisation, perceive a follow‐up care that is person‐centred and nurse‐led compared to standard care delivered by surgeons. The results indicate that patients find the person‐centred and nurse‐led follow‐up programme satisfactory, with equal or higher quality of care and that follow‐up can be delivered by nurses with retained patient safety. Thus, vascular units may consider transitioning follow‐up care from surgeons to nurses while maintaining positive patient's perception of quality of care, patient satisfaction and safety.

**Reporting Method:**

Reporting of the work was made using the Consolidated Standards of Reporting Trials (CONSORT) statement.

**Patient or Public Contribution:**

No patient or public contribution.

**Trial Registration:**

Study Details | Person‐centred Follow‐up and Health Promotion Programme After Revascularization for Intermittent Claudication | ClinicalTrials.gov: NCT03283358

**Summary**

- What does this paper contribute to the wider global clinical community?
  ○ The quality of person‐centred, nurse‐led follow‐up care is perceived as satisfactory among patients undergoing revascularisation for Intermittent Claudication.
  ○ Follow‐up of patients revascularised for Intermittent Claudication can be delivered by nurses without compromising patient safety.

## 1 Introduction

Intermittent claudication (IC) is a common symptom of peripheral artery disease and affects 7% of the population over 60 years of age (Sigvant et al. 2007). IC is associated with reduced health‐related quality of life due to negatively changed social and physical function, pain and impaired walking ability (Abaraogu et al. 2018; Aboyans et al. 2018). Patients with IC are referred to vascular units for surgical treatment when medical treatment, supervised exercise and lifestyle changes delivered by primary care are not enough (Aboyans et al. 2018). Post‐interventional follow‐up with risk factor modification and medical treatment is recommended after surgery (Aboyans et al. 2018).

## 2 Background

Evaluations of quality of care in complex intervention processes help caregivers and researchers in interpreting and understanding outcomes. The aim of evaluation can include exploring the views of care receivers/study participants on the given care/intervention and investigating the contextual aspects and components of the given care/intervention. Moreover, process evaluations can contribute to explaining for whom, how and why an intervention has a particular impact (Skivington et al. 2021). The Institute of Medicine defines good quality of care as the degree to which health services for individuals and populations increase the likelihood of desired health outcomes consistent with current professional knowledge (Institute of Medicine (US) The National Roundtable on Health Care Quality and Donaldson 1999). Good quality of care is not equal to good outcomes, since there are several aspects that affect outcomes other than just good quality of care. Health care provided in a timely manner, evidence‐based, effective, safe, patient‐focused and equitable, is a prerequisite for good care (Institute of Medicine (US) The National Roundtable on Health Care Quality 2001).

Patient reported experience measures (PREM) is information gathered on how patients view their experiences while receiving care. PREMs can be classified as either relational or functional and measure how the patient's experience is impacted by the process of the care. Relational PREMs examine the patients' experience of their encounter with the care provider during treatment, e.g., the feeling of being listened to and communicated with. Functional PREMs investigate more practical issues, such as the timeliness of assistance and the availability of facilities (Kingsley and Patel 2017). Thus, both patient centredness and the quality of delivered health care can be indicated by PREM (Bull et al. 2019; Hodson et al. 2013). Acquiring an increased understanding of how patients perceive the quality of care can contribute to an improved quality of care based on the patient's perspective (Wilde et al. 1994).

## 3 The Study

### 3.1 Aim

To describe and compare patients' perspectives on quality of care after receiving either person‐centred, nurse‐led follow‐up or standard care after surgical treatment of intermittent claudication.

## 4 Methods

### 4.1 Design

This study is a secondary analysis of a randomised controlled clinical trial, the Person‐centred Follow‐up After Surgical Treatment for Intermittent Claudication (FASTIC) study.

### 4.2 Study Setting and Sampling

**FIGURE 1:**
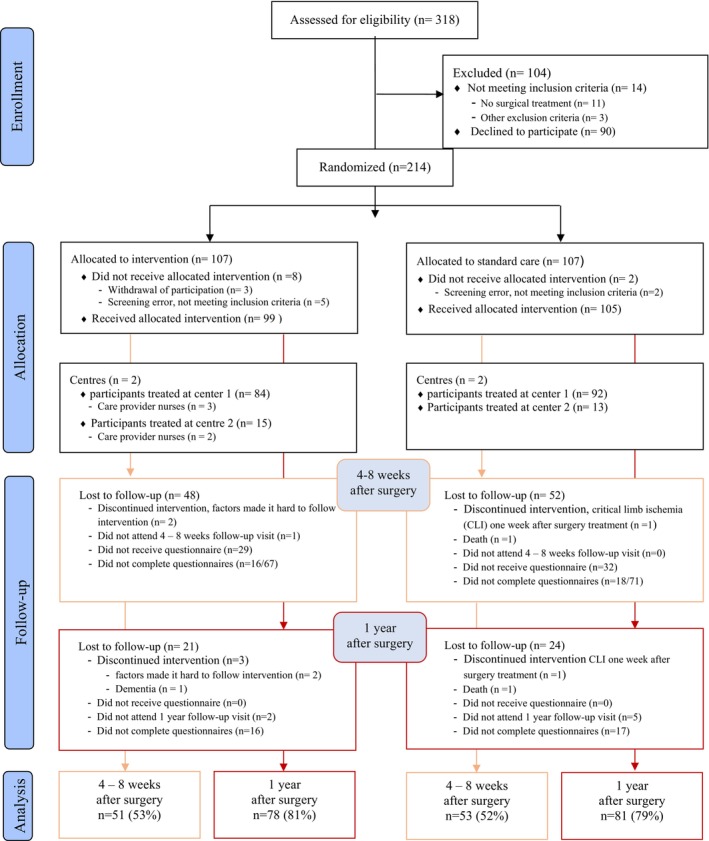
Modified CONSORT flow diagram for individual randomised controlled trials of nonpharmacologic treatments. CLI, critical limb ischemia; CONSORT, consolidated standards of reporting trials. [Colour figure can be viewed at wileyonlinelibrary.com]

The study was conducted from June 2016 to November 2019 at two centres for vascular surgery at two hospitals in Stockholm, Sweden. All eligible patients according to the FASTIC study's inclusion criteria who provided written informed consent were included and allocated to either a standard or person‐centred, nurse‐led follow‐up programme (Figure 1).

### 4.3 Sample Size

According to the researcher who developed the questionnaire, an item should be answered by at least 30 participants in order to avoid misinterpretation of statistical analyses or results (Wilde Larsson 2001). The sample size of 186 was calculated using the primary outcome for the FASTIC study (Haile et al. 2020, 2022a) was therefore regarded as sufficient for this study.

### 4.4 Inclusion and Exclusion Criteria

Inclusion criteria were adult patients over 18 years, diagnosed with IC, scheduled for surgical treatment and no sign of critical limb ischaemia. Exclusion criteria were patients diagnosed with dementia, planned discharge to a nursery home, not accountable for administering their own medication and a survival expectancy of less than 1 year.

### 4.5 Study Interventions

The intervention group received person‐centred and nurse‐led follow‐up with specially trained vascular nurses during three visits (4–8 weeks, 6 months and 1 year after surgery) and two telephone calls (2 and 9 months after surgery). The patients who received standard care follow‐up had two visits (4–8 weeks and 1 year after surgery) at the outpatient clinic. The patients met a vascular surgeon at the first visit and a vascular nurse at the second visit. The intervention group received person‐centred and nurse‐led follow‐up with specially trained vascular nurses during three visits (4–8 weeks, 6 months and 1 year after surgery) and two telephone calls (2 weeks and 9 months after surgery). A detailed protocol and content of the standard follow‐up programme as well as the person‐centred, nurse‐led follow‐up programme are described and published elsewhere (Haile et al. 2020).

### 4.6 Questionnaire

The questionnaire Quality from the Patient's Perspective (QPP) was used to measure quality of care from patients' perspective. This questionnaire was originally developed by Bodil Wilde‐Larsson et al. and is currently administered by Improveit^TM^. The QPP is based on patients' perceptions of care quality depending on their expectations, norm systems, experiences and encounters with caregivers. A holistic perspective is formed by assessing four interdependent dimensions (medical‐technical competence, physical‐technical conditions, identity‐oriented approach and socio‐cultural atmosphere) and associated sub‐items (Wilde et al. 1994; Wilde Larsson and Larsson 2002).

The questionnaire used in this study, based on the short version of QPP adapted for outpatient care, was modified with the assistance of and approved by ImproveIt. It consisted of 28‐items of four dimensions (three items of the dimension medical‐technical competence: one item of the dimension physical‐technical condition; 14 items of the dimension identity‐oriented approach; and 10 items of the socio‐cultural atmosphere). Each item was evaluated by the patients in two perspectives: (1) Perceived reality (PR), where the patients scored their experience “This is what I experienced…” from 1 (do not agree at all) to 4 (completely agree). (2) Subjective importance (SI), where the respondent scored the subjective importance ascribed to the item “This is how important it was to me…” from 1 (of little or no importance) to 4 (of the very highest importance). Each item had a response option “not applicable”.

In addition, the questionnaire included questions about level of education, confidence in getting information in Swedish, occupation, general physical health status and general psychological health status. The main outcome of the study was patient‐experienced quality of care (PR and SI) regarding the 28 items measured by the QPP.

### 4.7 Data Collection

After the visits at 4–8 weeks and 1 year after surgery, the patients were given the outpatient care version of the questionnaire QPP with prepaid and addressed envelopes. Initially, the study was designed to assess QPP only at 1‐year after surgery. After the study had been running for 6 months, it became apparent that it was only at the 4–8 week follow‐up visit that the control group actually met a surgeon. At the 1‐year visit, the control group was being scheduled with a non‐FASTIC nurse rather than with a surgeon. We therefore decided to start distributing the QPP questionnaire also at the 4–8 week visit, in order to investigate whether there were differences between patient experiences of quality of care depending on whether the patient met with a nurse or a surgeon. Thus, patients who had their visit prior to 5th of January 2017 were not included in the assessment at 4–8 weeks after surgery (Figure 1).

### 4.8 Data Analysis

The data was analysed with the IBM Statistical Package for Social Sciences (SPSS), version 28.0 (IBM Corp., Armonk, NY, USA). Categorical data is presented as frequencies (percentage), ordinal data as mean and median (IQR) and continuous not normally distributed data (age) as median (IQR). Depending on the data, either Fisher's exact test, Pearson chi‐square, or Mann–Whitney U test was used to compare cross‐sectional differences between groups at the two separate time points (4–8 weeks and 1 year after surgery). Two‐tailed *p‐*values of < 0.05 were considered statistically significant.

### 4.9 Ethical Considerations

The study was approved by the Regional Ethical Review Board in Stockholm (reference number 2015/2346‐31/2). All participants were given written and oral information about the study, and a written informed consent was required prior to inclusion in the study. The trial was conducted in compliance with the Helsinki Declaration (World Medical Association 2013) and reported in adherence to the Consolidated Standards of Reporting Trials (CONSORT) (File S1), Statement for Randomised Trials of Nonpharmacologic Treatment (Boutron et al. 2017).

## 5 Results

A total of 318 patients were assessed for eligibility and ninety out of those declined to participate. Eleven patients were not included because no surgical treatment was performed, and three were excluded due to other exclusion criteria. The remaining 214 patients were allocated to either the intervention group (*n* = 107) or the control group (*n* = 107). After randomisation, respectively five and two patients from the interventions and the control group were excluded due to screening error (not meeting inclusion criteria). Three additional patients in the intervention group were excluded due to withdrawal of consent. A total of 204 patients remained and received allocated intervention at centre one (*n* = 176) and at centre 2 (*n* = 28). Five of the patients did not fulfil the study protocol (Figure 1). At 4–8 weeks after surgery, 199 patients attended scheduled visit but only 138 received questionnaires. Of those, 51/67 in the intervention and 53/71 in the control group completed the questionnaires (Figure 1). There was no difference in characteristics between the groups (Table 1). The data showed that the population who did not receive questionnaires did not differ in characteristics when compared to those who received the questionnaires.

At 1‐year all (*n* = 193), except those not attended scheduled visit, received and 78 (81%) in the intervention group as well as 81 (79%) in the control group completed the questionnaires (Figure 1). The groups were comparable in characteristics except for a gender difference at 1‐year after surgery with significant more women in the control group compared to the intervention group (*p* = 0.026) (Table 1).

**TABLE 1:** Characteristics of the study population at 4–8 weeks and 1 year follow‐up after surgery for intermittent claudication.

	4–8 Weeks after surgery			1‐Year after surgery		
	Person‐centred, nurse‐led care, *N* = 51	Standard care, *N* = 53	*p*	Person‐centred, nurse‐led care, *N* = 78	Standard care, *N* = 81	*p*
Age, years	71 (65–78)	72 (68–76)	0.767	71 (66.75–76)	73 (69–76)	0.391
Female	18 (35)	27 (51)	0.118	29 (37)	45 (56)	0.026
Highest level of education^a^			0.801			0.781
College or university	17 (35)	19 (37)		23 (30)	28 (35)	
High school or upper secondary school	19 (39)	17 (33)		34 (44)	32 (40)	
Elementary school	13 (27)	16 (31)		20 (26)	20 (25)	
Occupation^b^			0.791			0.391
Employee	9 (19)	8 (15)		15 (20)	11 (14)	
Student	0 (0)	0 (0)		0 (0)	0 (0)	
Other	39 (81)	45 (85)		61 (80)	70 (86)	
Feel confident in getting information in Swedish^c^	49 (100)	53 (100)	NA	78 (100)	80 (99)	1.000
Physical health status^d^			0.325			0.336
Very good	12 (24)	19 (36)		17 (22)	22 (27)	
Relatively good	24 (47)	21 (40)		40 (51)	26 (32)	
Neither good nor bad	5 (10)	7 (13)		11 (14)	15 (19)	
Relatively poor	6 (12)	2 (4)		5 (6)	16 (20)	
Very poor	0 (0)	2 (4)		2 (3)	0 (0)	
Psychological health status^e^			0.213			0.308
Very good	21 (41)	28 (53)		38 (49)	32 (40)	
Relatively good	15 (29)	17 (32)		28 (36)	36 (44)	
Neither good nor bad	9 (18)	3 (6)		7 (9)	7 (9)	
Relatively poor	2 (4)	3 (6)		2 (3)	4 (5)	
Very poor	0 (0)	0 (0)		1 (1)	0 (0)	

*Note:* Values are presented as number (percentage) except for age presented as median (first quartile – third quartile). NA (non‐applicable).

^a^ Missing at 4–8 weeks *n* = 2|1; at 1 year *n* = 1|1.

^b^ Missing at 4–8 weeks *n* = 3|0; at 1 year *n* = 2|0.

^c^ Missing at 4–8 weeks *n* = 2|0.

^d^ Missing at 4–8 weeks *n* = 4|2; at 1 year *n* = 3|2.

^e^ Missing at 4–8 weeks *n* = 4|2; at 1 year *n* = 2|2.

### 5.1 Perceived Quality of Care and Subjective Importance at 4–8 Weeks After Surgery

Patients in the intervention group perceived the quality of care regarding information on self‐care “how I should take care of myself” significantly higher (*p* < 0.001) than the control group. The results also showed a higher perception in quality of care (*p* < 0.001) and subjective importance (*p* = 0.009) for the item “which nurse were responsible for my care”. Subjective importance for the item “nurses showed commitment/cared about me” was higher (*p* = 0.013) in the intervention group than the control group while there was no difference in the perceived quality of care for the same item. The item “opportunity to talk to the nurses in private” was scored higher both in perceived reality (*p* = 0.043) and in subjective importance (*p* = 0.043) among the intervention group.

### 5.2 Perceived Quality of Care and Subjective Importance at 1 Year After Surgery

The intervention group scored the perceived quality of care significantly higher than the control group for the item “which nurses were responsible for my care” (*p* = 0.007). For all other items, there were no significant differences between the groups regarding perceived quality of care. However, there were significant differences between the groups regarding subjective importance of the items with higher scores for the intervention group for six items:: information about “which nurses were responsible for my care” (*p* = 0.043), “effects and use of medicine” (*p* = 0.047), “nurses were respectful towards me” (*p* = 0.027), “nurses showed commitment/cared about me” (*p* = 0.031), “care was determined depending on my needs rather than caregivers routines” (*p* = 0.007) and “pleasant atmosphere at the clinic” (*p* = 0.021).

The items “effective pain relief”, “access to necessary apparatus and equipment” and many items in the dimension of the socio‐cultural atmosphere were perceived as non‐applicable by a two‐digit number of patients (Table 2, Table 3).

**TABLE 2:** Patients' perception of quality of care at 4–8 weeks after surgery for intermittent claudication.

Dimensions and items		Person‐centred, nurse‐led care, *N* = 51					Standard care, *N* = 53					*p*
		*n*	NA	Mean	Median	Q1–Q3	*n*	NA	Mean	Median	Q1–Q3	
Medical‐technical competence												
Best possible medical treatment	PR	43	5	3.9	4	4–4	50	2	3.7	4	4–4	0.510
	SI	40	5	3.7	4	3–4	45	2	3.6	4	3–4	0.827
Effective pain relief	PR	37	12	3.7	4	4–4	40	12	3.6	4	4–4	0.825
	SI	32	14	3.7	4	3–4	34	12	3.6	4	3–4	0.869
Examinations and treatment within acceptable waiting time	PR	46	2	3.7	4	4–4	51	1	3.7	4	4–4	0.753
	SI	40	2	3.5	4	3–4	43	1	3.4	3	3–4	0.309
Physical‐technical condition												
Access to necessary apparatus and equipment	PR	30	18	3.8	4	4–4	26	24	3.5	4	3–4	0.106
	SI	25	19	3.6	4	3–4	21	25	3.5	4	3–4	0.542
Identity‐oriented approach												
Receiving useful information about…												
…health status/disease	PR	50	0	3.7	4	3.75–4	52	0	3.7	4	4–4	0.924
	SI	46	2	3.6	4	3–4	49	0	3.6	4	3–4	0.861
…how examinations and treatments would take place	PR	50	0	3.6	4	3.75–4	52	0	3.7	4	4–4	0.653
	SI	46	1	3.5	4	3–4	45	2	3.6	4	3–4	0.229
…the results of examinations and treatments	PR	49	0	3.7	4	4–4	52	0	3.5	4	3–4	0.162
	SI	47	1	3.6	4	3–4	46	0	3.5	4	3–4	0.445
…self‐care. “How I should take care of myself”	PR	49	1	3.8	4	4–4	53	0	3.2	3	2–4	**< 0.001**
	SI	46	2	3.5	4	3–4	44	2	3.3	3	3–4	0.264
…which doctors were responsible for my care	PR	43	5	3.3	4	3–4	51	0	3.4	4	3–4	0.595
	SI	40	8	3.2	3	3–4	46	0	3.3	3.50	3–4	0.594
…which nurses were responsible for my care	PR	48	2	3.7	4	4–4	52	0	2.9	3	2–4	**< 0.001**
	SI	46	3	3.4	3.5	3.5–4	47	1	2.8	3	2–4	0.**009**
…effects and use of medicine	PR	43	6	3.5	4	3–4	45	7	3.3	4	3–4	0.212
	SI	40	7	3.5	4	3–4	42	7	3.3	4	3–4	0.232
Caregiver's understanding/empathy, respect and commitment												
Doctors seemed to understand my situation	PR	35	13	3.6	4	3–4	52	0	3.5	4	3–4	0.476
	SI	32	13	3.6	4	3–4	46	0	3.6	4	3–4	0.413
Nurses seemed to understand my situation	PR	49	0	3.8	4	4–4	50	2	3.6	4	3–4	0.061
	SI	42	0	3.6	4	3–4	44	2	3.5	4	3–4	0.417
Doctors were respectful towards me	PR	36	13	3.8	4	4–4	52	0	3.8	4	4–4	0.699
	SI	32	13	3.7	4	3–4	46	0	3.6	4	3–4	0.419
Nurses were respectful towards me	PR	50	0	4.0	4	4–4	51	1	3.8	4	4–4	0.**016**
	SI	43	0	3.7	4	3–4	46	1	3.6	4	3–4	0.336
Doctors showed commitment/cared about me	PR	35	13	3.6	4	3–4	52	0	3.6	4	4–4	0.826
	SI	33	13	3.6	4	3–4	46	0	3.6	4	3–4	0.962
Nurses showed commitment/cared about me	PR	50	0	3.9	4	4–4	51	1	3.7	4	3–4	0.**013**
	SI	43	0	3.6	4	3–4	46	1	3.5	4	3–4	0.216
Had opportunity to participate in the decisions applied to my care	PR	42	8	3.5	4	3–4	35	16	3.3	4	3–4	0.439
	SI	33	10	3.5	4	3–4	28	18	3.4	3	3–4	0.433
Socio‐cultural atmosphere												
Care was determined depending on my needs rather than care givers routines	PR	42	5	3.6	4	3–4	43	8	3.3	3	3–4	0.066
	SI	37	5	3.4	4	3–4	38	9	3.3	3	3–4	0.302
Next of kin treated well	PR	15	29	3.8	4	4–4	25	25	3.6	4	3–4	0.659
	SI	14	29	3.4	3.5	3–4	20	26	3.6	4	3–4	0.416
Pleasant atmosphere at the clinic	PR	50	1	3.7	4	3.75–4	52	0	3.7	4	4–4	0.563
	SI	42	1	3.6	4	3–4	44	0	3.7	4	3–4	0.389
Opportunity to talk to the doctor in private	PR	20	29	3.1	3	3–4	26	23	3.1	3.50	2–4	0.981
	SI	18	30	3.3	3	3–4	20	25	2.9	3	2–3.75	0.119
Opportunity to talk to the nurse in private	PR	35	15	3.5	4	3–4	24	24	3.1	3	2.25–4	0.**043**
	SI	29	16	3.5	4	3–4	19	26	3.1	3	3–4	0.**034**
Easy to get in contact with the clinic through telephone	PR	24	24	3.5	4	3–4	31	20	3.1	3	2–4	0.059
	SI	21	25	3.4	3	3–4	30	21	3.0	3	2–4	0.079
Easy to get in contact with the doctor through telephone	PR	13	35	2.4	2	1–3.5	19	30	2.8	3	2–4	0.362
	SI	12	35	3.0	3	2.25–3.75	16	31	3.1	3	2.25–4	0.732
Easy to get in contact with the nurse through telephone	PR	21	27	3.5	4	3–4	23	26	3.2	3	3–4	0.262
	SI	18	27	3.2	3	3–4	21	27	3.2	3	3–4	0.770
Easy to schedule a visit with a doctor	PR	20	28	3.1	3	2.25–4	26	24	3.0	3	2–4	0.707
	SI	19	28	3.4	4	3–4	24	25	3.3	3	3–4	0.679
Easy to schedule a visit with a nurse	PR	24	23	3.5	4	3–4	19	30	3.2	3	3–4	0.114
	SI	21	23	3.5	4	3–4	17	31	3.2	3	2.50–4	0.416

*Note:* PR = perceived reality, scores could range from 1 (do not agree at all) to 4 (completely agree) or an alternative non‐applicable; SI = subjective importance, scores could range from 1 (of no or little importance) to 4 (of a highest importance) or an alternative non‐applicable; *n* = number of respondents with score 1–4 for each item; NA = number of respondents who answered “non‐applicable”; Q1; Q3 = First quartile; third quartile. Significant differences between groups (two‐tailed *p*‐value) are presented in bold font. Mann–Whitney U test was used to compare differences between groups.

**TABLE 3:** Patients' perception of quality of care at 1 year after surgery for intermittent claudication.

Dimensions and items		Person‐centred, nurse‐led care, *N* = 78					Standard care, *N* = 81					*p*
		*n*	NA	Mean	Median	Q1–Q3	*n*	NA	Mean	Median	Q1–Q3	
Medical‐technical competence												
Best possible medical treatment	PR	72	3	3.8	4	4–4	74	7	3.7	4	3.75–4	0.385
	SI	65	5	3.7	4	3–4	66	8	3.5	4	3–4	0.051
Effective pain relief	PR	43	17	3.7	4	3.5–4	49	28	3.6	4	3.5–4	0.975
	SI	49	19	3.5	4	3–4	46	29	3.5	4	3–4	0.943
Examinations and treatment within acceptable waiting time	PR	71	3	3.6	4	3–4	77	2	3.7	4	4–4	0.349
	SI	64	3	3.5	4	3–4	70	4	3.4	3	3–4	0.143
Physical‐technical condition												
Access to necessary apparatus and equipment	PR	39	31	3.8	4	4–4	41	36	3.5	4	3–4	0.058
	SI	31	33	3.6	4	3–4	36	38	3.5	4	3–4	0.363
Identity‐oriented approach												
Receiving useful information about…												
Health status/disease	PR	75	0	3.8	4	4–4	76	2	3.7	4	3.25–4	0.340
	SI	71	0	3.5	4	3–4	74	3	3.4	3.5	3–4	0.295
How examinations and treatments would take place	PR	75	0	3.8	4	4–4	77	1	3.7	4	4–4	0.496
	SI	70	0	3.6	4	3–4	72	4	3.4	4	3–4	0.350
The results of examinations and treatments	PR	75	0	3.7	4	4–4	77	1	3.6	4	3–4	0.561
	SI	70	0	3.6	4	3–4	72	3	3.4	4	3–4	0.271
Self‐care. “How I should take care of myself”	PR	75	0	3.6	4	3–4	77	1	3.3	4	3–4	0.093
	SI	69	1	3.4	4	3–4	72	4	3.1	3	3–4	0.059
Which doctor were responsible for my care	PR	62	8	3.2	3.5	3–4	74	3	3.1	4	2–4	0.700
	SI	59	9	3.3	3	3–4	66	8	3.0	3	2–4	0.210
Which nurses were responsible for my care	PR	70	3	3.7	4	4–4	74	4	3.3	4	3–4	0.**007**
	SI	64	4	3.5	4	3–4	64	9	3.2	3	3–4	0.**043**
Effects and use of medicine	PR	67	6	3.6	4	3–4	74	7	3.5	4	3–4	0.462
	SI	62	7	3.6	4	3–4	67	10	3.3	4	3–4	0.**047**
Caregiver's understanding/empathy, respect and commitment												
Doctors seemed to understand my situation	PR	57	13	3.6	4	3–4	59	15	3.4	4	3–4	0.244
	SI	51	15	3.7	4	3–4	64	17	3.5	4	3–4	0.086
Nurses seemed to understand my situation	PR	72	2	3.7	4	4–4	76	4	3.7	4	3.25–4	0.453
	SI	66	2	3.6	4	3–4	65	5	3.4	4	3–4	0.116
Doctors were respectful towards me	PR	61	12	3.7	4	4–4	64	15	3.7	4	3–4	0.393
	SI	54	14	3.6	4	3–4	57	16	3.5	4	3.25–4	0.222
Nurses were respectful towards me	PR	73	2	3.9	4	4–4	75	4	3.8	4	4–4	0.198
	SI	67	2	3.7	4	3.7–4	66	5	3.5	4	3–4	0.**027**
Doctors showed commitment/cared about me	PR	59	13	3.7	4	3–4	65	15	3.5	4	3–4	0.393
	SI	51	15	3.6	4	3–4	58	16	3.5	4	3–4	0.222
Nurses showed commitment/cared about me	PR	73	2	3.8	4	4–4	75	4	3.7	4	4–4	0.284
	SI	67	2	3.7	4	3–4	64	5	3.4	4	3–4	0.**031**
Had opportunity to participate in the decisions applied to my care	PR	63	11	3.5	4	3–4	64	13	3.3	4	3–4	0.061
	SI	53	14	3.6	4	3–4	56	15	3.3	4	3–4	0.110
Socio‐cultural atmosphere												
Care was determined depending on my needs rather than care givers routines	PR	66	6	3.5	4	3–4	61	13	3.4	4	3–4	0.321
	SI	55	9	3.6	4	3–4	57	14	3.3	3	3–4	0.**007**
Next of kin treated well	PR	28	42	3.6	4	4–4	33	36	3.7	4	4–4	0.058
	SI	23	45	3.7	4	3–4	29	38	3.6	4	3–4	0.363
Pleasant atmosphere at the clinic	PR	73	2	3.7	4	3–4	76	3	3.5	4	3–4	0.156
	SI	64	2	3.5	4	3–4	70	5	3.2	3	3–4	0.**021**
Opportunity to talk to the doctor in private	PR	37	33	3.1	3	2.5–4	38	42	3.4	4	3–4	0.295
	SI	28	39	3.5	4	3–4	34	45	3.3	3	3–4	0.336
Opportunity to talk to the nurse in private	PR	54	20	3.6	4	3–4	42	37	3.5	4	3–4	0.896
	SI	43	25	3.5	4	3–4	36	39	3.4	3.5	3–4	0.240
Easy to get in contact with the clinic through telephone	PR	35	36	3.6	4	3–4	48	28	3.5	4	3–4	0.378
	SI	27	36	3.6	4	3–4	43	28	3.3	3	3–4	0.061
Easy to get in contact with the doctor through telephone	PR	19	50	3.0	3	2–4	28	47	2.7	3	2–3.75	0.319
	SI	14	50	3.4	4	2.75–4	24	48	3,1	3	2.25–4	0.361
Easy to get in contact with the nurse through telephone	PR	27	41	3.5	4	3–4	34	39	3.4	4	3–4	0.829
	SI	21	41	3.6	4	3–4	29	41	3.3	3	3–4	0.170
Easy to schedule a visit with a doctor	PR	31	40	3.3	4	3–4	41	36	3.2	3	3–4	0.882
	SI	25	40	3.5	4	3–4	35	36	3.4	4	3–4	0.484
Easy to schedule a visit with a nurse	PR	35	40	3.7	4	3–4	34	44	3.5	4	3–4	0.272
	SI	28	41	3.7	4	3.25–4	27	45	3.4	4	3–4	0.078

*Note:* PR = perceived reality, scores could range from 1 (do not agree at all) to 4 (completely agree) or an alternative non‐applicable; SI = subjective importance, scores could range from 1 (of no or little importance) to 4 (of a highest importance) or an alternative non‐applicable; *n* = number of respondents with score 1–4 for each item; NA = number of respondents who answered “non‐applicable”; Q1; Q3 = First quartile; third quartile. Significant differences between groups (two‐tailed *p*‐value) are presented in bold font. Mann–Whitney U test was used to compare differences between groups.

## 6 Discussion

The study shows that the intervention group who received person‐centred, nurse‐led follow‐up demonstrated a higher perception of quality of care regarding information about self‐care, experience of being respected and of knowing the care provider responsible for their care compared to the control group. The intervention group scored significantly higher perceived quality of care regarding three items in the identity‐oriented approach: “How I should take care of myself”, “Which nurses were responsible for my care” and “Nurses showed commitment/cared about me”. The control group scored subjective importance higher than the perceived reality for the item “self‐care”, which indicates the need for more information on self‐care than received. The importance of providing patients with information about self‐care, the disease, treatment and secondary prevention is emphasised in the literature (Gorely et al. 2015; Harwood et al. 2017).

Increased knowledge about disease and self‐care has been suggested to facilitate adherence to treatment by improving the patients' understanding of why they should follow the recommendations given to them by the healthcare provider (Abaraogu et al. 2018; Aherne et al. 2017). The person‐centred and nurse‐led follow‐up programme applied in this study has earlier been reported to facilitate maintaining lifestyle changes among patients surgically treated for IC (Haile et al. 2022b).

One‐year after surgery, significant difference regarding perceived reality and subjective importance was noted only for one item “which nurses were responsible for my care”. Perceived quality of care regarding subjective importance was higher in the intervention group for the items: “nurses were respectful towards me”, “effects and use of medicine”, “nurses showed commitment/cared about me”, “care was determined depending on my needs rather than caregivers' routines” and “pleasant atmosphere at the clinic”. Nevertheless, no differences were noted regarding perceived reality. The difference shown in the result regarding subjective importance for the items “which nurses were responsible for my care”, “nurses were respectful towards me” and “opportunity to talk to the nurse in private” could be due to the nurses' role in the standard follow‐up program is merely assisting the doctor.

At the 1‐year follow‐up, the patients in both groups were scheduled to meet a nurse. The difference was that the intervention group met the FASTIC nurses they had met throughout the follow‐up year whereas the control group met a non‐FASTIC nurse per standard routine. It could be possible that the higher perception regarding subjective importance was a reflection of the care given throughout the year and not the care during a single visit. A study that investigated the association between surgical patient satisfaction and non‐modifiable factors, using outpatient satisfaction scores from 18,373 completed surveys at an academic department of surgery reported that younger patients and patients who see their healthcare provider for the first time were less likely to report complete satisfaction. Patients seen in the vascular surgery clinics were also less likely to be satisfied when compared with general surgery clinics (Martin et al. 2017). Regardless follow‐up programme, the patients had lower scores in perceived reality than subjective importance for the items “easy to get in contact with the doctor through telephone” and “easy to schedule a visit with a doctor” at both measuring times. According to the guidelines to the questionnaire QPP (Wilde Larsson 2001), a high score in subjective importance with a low index in perceived reality indicates insufficiency in quality of care and need for improvement (Wilde Larsson 2001) in aspect of those items.

### 6.1 Strengths and Limitations

The high response rate of the questionnaires is a strength of this study. One limitation of the study is the delayed start of the distribution of questionnaires at 4–8 weeks after surgery, which led to discrepancies between the larger sample size (159 questionnaires) at the one‐year follow‐up and the smaller sample size (104 questionnaires) at the 4–8‐week follow‐up. Nonetheless, as there were no significant demographic differences between the participants who received the questionnaire at 4–8 weeks and the participants who did not, it can be assumed that this did not affect the results of the study. There was an overrepresentation of females in the control group among the analysed population at the 1‐year follow‐up, which could have impacted the result. Although it has been reported that gender has no significant role in scoring the perceived reality of quality of care using QPP, females tend to score higher subjective importance than males (Martin et al. 2017). Accordingly, if the gender distribution had been the same in both groups, there might have been more items regarding subjective importance with significant differences in favour of the intervention group. Another limitation of the study is that it was conducted in two specialised clinics and on a homogenous patient group and did not address the adaptability of the intervention programme in other healthcare systems with different healthcare settings, staffing limitations, resources and/or patient groups.

### 6.2 Recommendations for Further Research

In addition to a cost analysis of the two follow‐up programmes, studies on the feasibility of person‐centred, nurse‐led follow‐up programmes in other contexts than specialised clinics are warranted. Further research may also be needed to assess nurse‐led care for patients with IC from a nurse's perspective.

### 6.3 Implications for Policy and Practice

The fact that the nurse‐led follow‐up programme can be delivered safely may lead to increased team‐based care or task shifting from surgeons to nurses.

### 6.4 Conclusion

Person‐centred, nurse‐led follow‐up as implemented in this study has shown to lead to a higher perception of quality of care regarding information about self‐care, experience of being respected and of knowing the caregiver responsible for their care. Thus, it could contribute towards patient satisfaction and person‐centred care without compromising the perception of quality of care regarding other factors, for example, receiving best medical care or timeliness.

## Author Contributions

**Sara Tibebe Haile** was involved in managing and coordinating the research activity planning and execution, conception and design, analysis and interpretation of data, drafting and revising of the manuscript and final approval of the version to be submitted. **Mina Olsson and Ronnie Lindstrand** were involved in making substantial contributions to data collection and analysis, revising the manuscript and final approval of the version to be submitted. **Helena Lööf** was involved in making substantial contributions to the analysis and interpretation of data, revising the manuscript, and final approval of the version to be submitted. **Unn‐Britt Johansson** made a substantial contribution to the conception and design, analysis and interpretation of data, revising the manuscript and final approval of the version to be submitted. **Anneli Linné** contributed to the conception and design, analysis and interpretation of data and final approval of the version to be submitted. **Eva Joelsson‐Alm** was involved in the conception and design, supervising the study, analysis and interpretation of data, drafting and revising the manuscript and final approval of the version to be submitted. Agreement to be accountable for all aspects of the work in ensuring that questions related to the accuracy or integrity of any part of the work are appropriately investigated and resolved.

## Conflicts of Interest

The authors declare no conflicts of interest.

## Supporting information


Data S1.

## Data Availability

The data that support the findings of this study are available on request from the corresponding author. The data are not publicly available due to privacy or ethical restrictions.
